# Development of a Robust Platform for Infrared Ion Spectroscopy: A New Addition to the Analytical Toolkit for Enhanced Metabolite Structure Elucidation

**DOI:** 10.1021/acs.analchem.5c03593

**Published:** 2025-07-31

**Authors:** Teun van Wieringen, Arnaud Lubin, Rianne van Outersterp, Jonathan Martens, Eric van Beelen, Jos Oomens, Filip Cuyckens, Giel Berden

**Affiliations:** † 6029 Radboud University, Institute for Molecules and Materials, FELIX Laboratory, Toernooiveld 7, Nijmegen 6525 ED, The Netherlands; ‡ Drug Metabolism & Pharmacokinetics, 81828 Johnson & Johnson, Turnhoutseweg 30, Beerse B-2340, Belgium; § 39118 Bruker Daltonics GmbH & Co. KG, Fahrenheitstraße 4, Bremen D-28359, Germany

## Abstract

Metabolite identification is essential in drug metabolism and pharmacokinetics (DMPK) studies and plays a pivotal role throughout the drug development process, from informing drug design to evaluating safety and efficacy. Mass spectrometry (MS) is the analytical technique of choice for characterizing metabolites due to its selectivity and sensitivity, particularly when paired with chromatographic methods. However, MS encounters challenges in structural characterization. This study employs infrared ion spectroscopy (IRIS) to differentiate isomeric compounds and demonstrates the robustness of a newly developed IRIS platform. We showcase applications in metabolite identification by determining the site of glucuronidation and phase I oxidation in selected drug molecules. Employing density functional theory for spectral prediction, IRIS decreases reliance on reference standards and alleviates the time-consuming purification processes typically associated with metabolite analysis. The newly developed platform integrates a high-power, high-repetition-rate infrared laser and ion trap MS. This setup is very robust, as evidenced by the highly reproducible IRIS spectra recorded over a one-year period without any instrument readjustment or recalibration. Moreover, the high power and high repetition rate of the laser provide a large dynamic range that is necessary to resolve all spectral features. These results leverage IRIS toward a transformative tool in analytical chemistry, with potential applications expanding across various fields, such as impurity analysis and forensics. The introduction of a compact IRIS setup in an industrial setting not only confirms its practical applicability but also emphasizes its potential for integration into routine analytical workflows.

## Graphical abstract

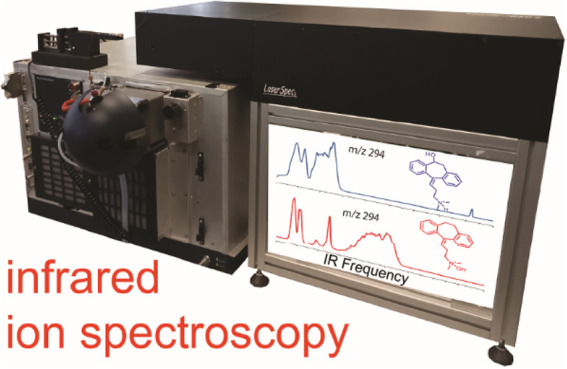

## Introduction

Metabolite identification is an essential and routine activity performed within drug metabolism and pharmacokinetics (DMPK) departments during the drug discovery and development process.^1,2^ From enhancing our understanding of biotransformation pathways to guide drug design in the discovery phase to assessing safety and efficacy in the development phase, metabolite identification plays a crucial role throughout the drug development process. When hyphenated with chromatographic techniques, mass spectrometry (MS) has established itself as the analytical technique of choice for characterizing metabolites due to its high selectivity and sensitivity.^3^

Despite its strengths in determining molecular composition, mass spectrometry often faces challenges in structural characterization. Tandem mass spectrometry (MS^n^) is available to provide a level of differentiation for certain isomeric compounds based on their unique fragmentation patterns. However, it frequently falls short of delivering complete structural elucidation, necessitating orthogonal analytical techniques.^4^ In such instances, nuclear magnetic resonance (NMR) spectroscopy is frequently favored, as it is able to define the exact position of a biotransformation within a drug molecule.^5^ Nonetheless, NMR also presents its own limitations. Despite recent advances, including the development of cryoprobes,^6^ several micrograms of purified analyte are still essential for successful structural identification. The complexities of biological matrices, whether derived from in vivo or in vitro methods, further complicate the purification process for NMR analysis, rendering it labor-intensive and time-consuming.

As alternative approaches, derivatization reactions coupled with liquid chromatography–mass spectrometry and advanced fragmentation techniques such as electron activated dissociation (EAD) or electrochemical metabolite generation have provided promising pathways.^4,7,8^ In this manuscript, we demonstrate how infrared ion spectroscopy (IRIS) can effectively address some of the challenges that mass spectrometry encounters in metabolite identification. This innovative technique offers a complementary strategy for metabolite characterization, enhancing the overall capabilities of the analytical toolkit available to researchers in the field.

IRIS is a technique that combines the selectivity and sensitivity of mass spectrometry (MS) with the structural information gained from IR spectroscopy.^9^ Based on MS^n^, IRIS employs a wavelength-tunable IR laser to induce fragmentation only when its emission wavelength is resonant with a vibrational absorption band of the mass-isolated ion population. By monitoring the fragmentation yield while scanning the wavelength of the laser, a vibrational fingerprint spectrum is obtained. IRIS has been shown to be able to determine the structure of a wide variety of organic molecules, such as human^10–12^ and plant metabolites,^13,14^ medicinal drug metabolites^15,16^ and glycans.^17–22^ IRIS is usually combined with computational chemistry^23^ to predict IR fingerprints, so as to enable identification of compounds for which reference standards are not available.

Most analytical IRIS experiments have been performed at large-scale user facilities with IR free electron lasers (FELs) that provide high power laser pulses over a wide wavelength range (2.7–50 μm, 200–3700 cm^–1^).^9,10,14,15,24–33^ A part of this spectral range (roughly between 2800 and 3800 cm^–1^) is also accessible with table-top laser systems, which have been connected to MS platforms by several academic groups.^13,34–39^ This spectral range covers the C–H, O–H and N–H stretching vibrations that often provide detailed structural information so that small molecule identification is possible.

Depending on the output power of the laser available, different strategies to implement IR ion spectroscopy can be chosen. When laser power is limited, tagging spectroscopy, where a weakly bound complex of the ion of interest is dissociated instead of the ion itself, is perhaps the best option,^19,35^ although it can only be applied in cryogenic mass spectrometers, which are not (yet) widely available from commercial MS manufacturers. If one wants to make use of a standard room temperature MS platform, a laser with high output power is required to efficiently induce infrared multiple-photon dissociation^40^ (IRMPD) at wavelengths resonant with molecular absorptions in the ion. Recently, we have demonstrated that high repetition rate (20 kHz–80 MHz) IR lasers provide a substantial improvement in IRMPD yield compared to both continuous-wave (cw) lasers and low repetition rate (10 Hz) lasers.^41^ Nevertheless, some ionic systems remain difficult to fragment with IR lasers, leading to low spectral band intensities or even missing features in their IR spectra, which is usually due to insufficient laser power.

In order to enable ion spectroscopy to take the leap from the academic to the industrial analytical laboratory, it is important that the IRIS platform is robust and capable of recording reproducible IR spectra of a large variety of ionic systems, without extensive day-to-day optimization and tuning. Here, we describe the newly developed very compact and stable IRIS platform in the analytical laboratory of Johnson & Johnson, benchmark its performance and give examples of analytical challenges that benefit from this platform.

## Experimental Section

Infrared Ion Spectroscopy measurements were performed with an infrared optical parametric oscillator (OPO) (LaserSpec, Belgium) connected to a 3D quadrupole ion trap (QIT) mass spectrometer with optical access to the trapped ion population (Bruker amaZon speed ETD), see Figure 1. All optical components, the OPO and its pump laser with electronics, are fully integrated with dimensions 890 (length) × 400 (width) × 180 (height) mm and mounted above the mass spectrometer. The IR frequency is tunable between 2800 and 3700 cm^–1^ (2.70–3.57 μm), and the bandwidth is 2 cm^–1^. The time-averaged OPO output power is 2 W. The laser pulses have a duration of approximately 10 ns and the time between the pulses is 33 μs (30 kHz repetition rate). This corresponds to a pulse energy of 67 μJ/pulse and a peak power of 6.7 kW.

**Figure 1:**
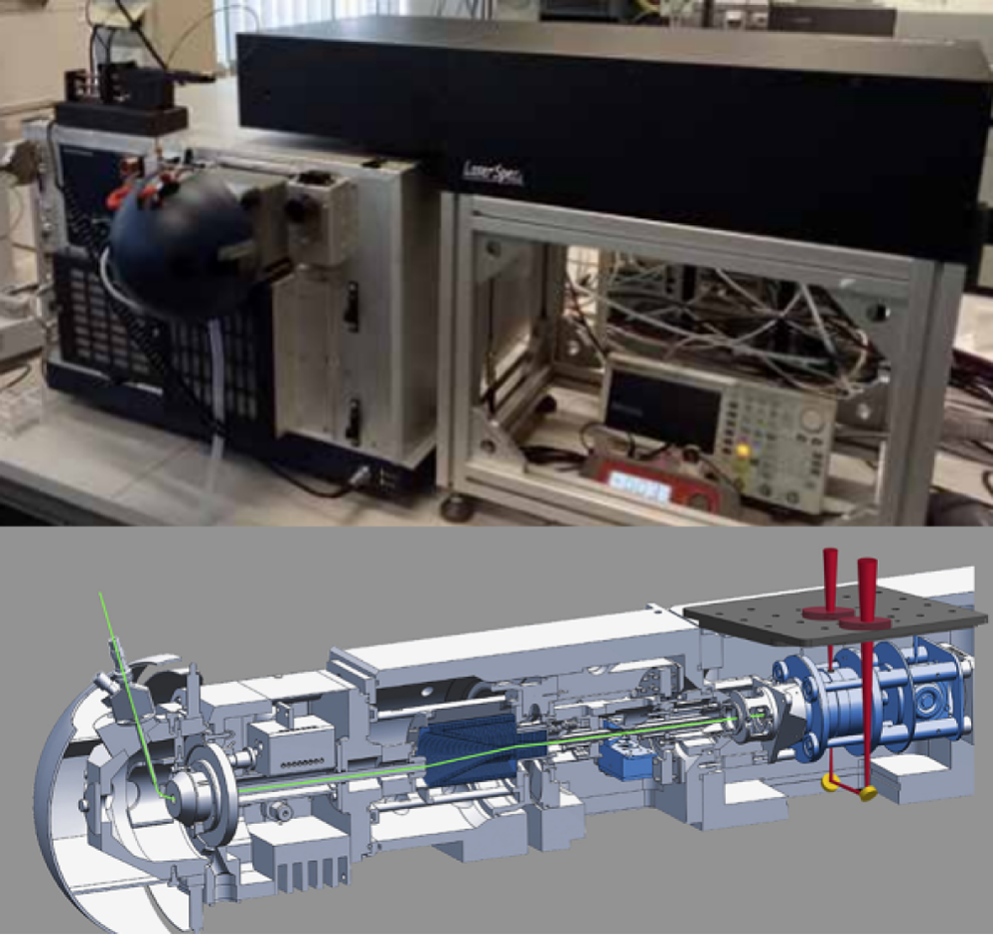
Top: photograph of the setup with the MS on the left with the ESI source at the front, and to the right, the frame with the black box being the IR laser. Bottom: schematic drawing of the ion trap MS with optical access to the trapped ions (reproduced with permission from ref 12. Copyright 2017 Springer Nature).

Inside the laser system, the OPO output beam is guided through a mechanical shutter (Thorlabs Optical Shutter SH05) triggered by the control electronics of the quadrupole ion trap^24^ allowing the operator to set the IR irradiation time of the trapped ions. The beam is focused using a CaF_2_ lens (see below) and directed downward into the MS. Two CaF_2_ windows (diameter 25 mm, clear aperture 16 mm) are mounted on top of the vacuum housing above the ion trap. The ring electrode of the trap contains two 3 mm holes centered at its top and bottom. Two gold-coated mirrors below the trap (see Figure 1) guide the IR laser beam via the exit window back into the laser box and onto a power meter. Via a dichroic mirror in front of the power meter, a 532 nm diode laser alignment beam is counterpropagating with the IR OPO beam through the MS. The laser is flushed with N_2_ to prevent IR absorption by atmospheric gases (especially water vapor). The position and focal length of the CaF_2_ lens was optimized to achieve maximum IR-induced ion fragmentation.

The MS was operated with a positive or negative ion electrospray ionization (ESI) source and dissolved samples (∼1 μM in 50:50 methanol:water) were infused at a 180 μL/h flow rate. In the ion trapping region, mass-to-charge (*m*/*z*) selected ions were irradiated by the wavelength-tunable infrared OPO to induce wavelength-dependent IRMPD, while scanning over the frequency range in steps of 3 cm^–1^.^9,42^ The high repetition rate of the laser mitigates the need for precise synchronization between laser pulses and the MS sequence and the trapped ions are irradiated for 27 to 1000 ms (810–30000 pulses) by triggering the mechanical shutter, which has a minimal opening time of 27 ms.^41^

IR spectra of *m*/*z*-selected ions are obtained by plotting the IRMPD yield as a function of IR frequency. The IRMPD yield is defined as the ratio of the sum of all fragment ions over the sum of all ions (fragments + precursor) and was obtained from 6 averaged mass spectra for each IR wavelength. The IR frequency was calibrated by recording the IR spectrum of protonated tryptophan using the strongest vibrational band at 3555 cm^–1^.^43^ Additionally, precursor ion depletion curves were obtained by recording the normalized precursor ion intensity (1 – IRMPD yield) as a function of irradiation time using an automated protocol described in reference^28^. Communication between laser and mass spectrometer is provided by a LabView program that also stores the IR frequency value into the Bruker MS data file.^24^

The ion trap employs a helium buffer gas for efficient trapping of ions. However, the buffer gas competes with the IRMPD process as collisions quench vibrational energy buildup during the IR excitation, reducing the IRMPD yield or even preventing dissociation entirely. Previously, we have shown that especially for high-repetition IR lasers, the IRMPD yield can be enhanced by reducing the buffer gas pressure.^41^ In the current setup, the He pressure can be adjusted as a percentile setting of the gas controller, here indicated as GC_He_. The standard setting for the instrument used here (calibrated for optimal ion signal) is 60%, which gives a pressure of ∼10^–3^ mbar in the trap. Lowering the GC_He_ to a value of 5–15% decreases the pressure to ∼10^–5^ mbar, while a value of 0% (no helium) gives a pressure of ∼10^–6^ mbar.

## Results and Discussion

### Benchmarking

In order to evaluate the performance of the new IRIS setup, we performed several experiments to benchmark the system against similar setups reported in literature. Protonated tryptophan ([Trp+H]^+^, *m*/*z* 205) is commonly employed for assessing the effectiveness of IRMPD spectroscopy in quadrupole ion traps.^24,41,43–45^ Within the spectral range of 3300–3600 cm^–1^, its IR spectrum contains a strong carboxylic acid OH stretching mode at 3555 cm^–1^, a prominent indole NH stretching mode at 3500 cm^–1^ and a weaker band associated with the NH_3_^+^ moiety at 3340 cm^–1^. The blue trace in Figure 2A shows the IR spectrum obtained with an irradiation time of 50 ms. The flat top of the strongest absorption band (OH stretch, 3555 cm^–1^) shows that the ion population is completely depleted. The band at 3500 cm^–1^ has a yield of 0.996 indicating that 0.4% of the ions remain undissociated. Modestly increasing the irradiation time to 200 ms (Figure 2A, red curve) makes the weaker vibrational bands more pronounced.

**Figure 2:**
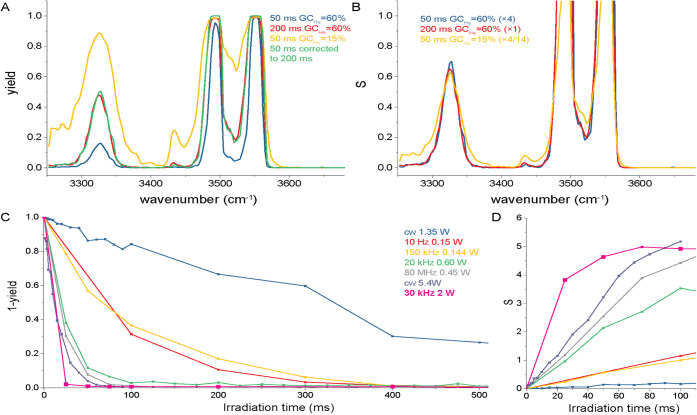
A: IRIS spectrum of protonated tryptophan for varying IR laser irradiation times and helium pressures. B: Alternative presentation of the same data where the vertical axis labeled *S* is the IRMPD intensity (see text). The parameter *S* is linearly scaled to account for the difference in laser irradiation time and helium pressure, making the spectra identical. C: Normalized precursor ion depletion (=1 – yield) as a function of irradiation time recorded using seven different OPO laser sources at 3555 cm^–1^ and normal helium pressures (GC_He_ = 60%). D: The same data as panel C with the vertical axis converted to *S*.

Instead of increasing the irradiation time, the helium pressure in the trap can be reduced to improve the dissociation yield, as shown by the yellow curve in Figure 2A (irradiation time 50 ms, GC_He_ = 15%). The higher dissociation yield is due to the significantly reduced collisional quenching rate relative to the constant photon absorption rate. Lowering the helium pressure, however, reduces the ion signal and hence sensitivity of the MS, so that studies targeting low-abundance compounds may become impractical. For a more in-depth discussion the reader is referred to ref. 41

The IR dissociation yield as defined here has a value between 0 (no dissociation) and 1 (complete dissociation). For comparison with calculated IR spectra and with precursor depletion spectra, the fragment fluence *S* = −ln­(1 – yield) is more appropriate as it more directly links to the photodissociation rate and therefore scales linearly with the irradiation time.^42,46,47^ In order to quantitively compare the three IR spectra, we have plotted *S* multiplied by a factor *C*, where *C* = 1 for 200 ms irradiation time and normal helium, *C* = 4 for 50 ms and normal helium, and *C* = 4/14 for 50 ms and reduced helium (Figure 2B). The two spectra recorded at normal helium pressure coincide perfectly, illustrating that the spectral intensity scales linearly. Comparing the two 50 ms spectra shows that for protonated Trp, the signal increases by a factor of 14 due to the reduced helium pressure. More importantly, Figure 2B demonstrates that after scaling, the spectra are identical, thus showing that under the current irradiation parameters (time and power), the experiment is conducted in the linear regime, well above the typical threshold that is often encountered in IRMPD studies.^42^

For benchmarking with results reported in literature, we recorded the normalized precursor ion intensity (= 1 – yield) and the fragment fluence *S* as a function of irradiation time while exciting the OH stretch mode. Note that the fragment fluence *S* is identical to −ln­(*I*_p_), where *I*_p_ is the normalized precursor ion intensity. The results are displayed in Figure 2C and D together with data reported in literature.^24,41,45^ High peak-power, low repetition-rate lasers, such as the 10 Hz systems with powers of around 150 mW,^41^ are popular for IRIS. Irradiation with a single laser pulse corresponds to 6 ns of irradiation time (the duration of the laser pulse), however, we plot the corresponding data point at 100 ms (in fact, with a single pulse, the irradiation time is limited by the mechanical shutter, about 30 ms). The 80 MHz 450 mW and 20 kHz 600 mW OPO (both from LaserSpec) data is taken from reference 41. The cw-OPO data (1350 mW and 5400 mW, Aculight Argos) is from reference 24 (another type of shutter was used allowing for shorter irradiation times). All experiments were carried out on a Bruker amaZon ion trap mass spectrometer. For completeness, we include data recorded using a linear ion trap (ThermoFinnegan LTQ XL) in combination with a 150 kHz 144 mW OPO (M Squared Firefly).^45^

Figure 2D displays *S* as a function of irradiation time, which clearly shows that for all laser systems the dependence is linear up to an IRMPD yield of 0.98 (*S* = 4). The highest IRMPD yields are expected for lasers with high peak powers, so short pulses with high pulse energy, and with high repetition rate, as a shorter time between laser pulses reduces the energy loss due to collisions with the buffer gas.^41^ The lasers used to obtain the data reported in Figure 2C and D are commercially available but present a trade-off between properties. What Figure 2C and D clearly show, is that the high-power 30 kHz laser used in the present study achieves the highest IRMPD yield.

Excellent repeatability and reproducibility of IRIS measurements are important aspects for the acceptance of the technique in an (pharmaceutical) industrial setting. Figure 3A shows four repeated measurements of protonated tryptophan on four different days over a span of 8 months. No adjustments to the laser, the QIT or the optical overlap with the ion cloud were made during this period. The data as displayed is raw: the wavenumbers are reported by the laser (i.e., without additional calibration) and the intensities are unscaled. Deviations from the mean value scale with the intensity (yield), see Figure 3B. Larger deviation is observed in the 3450–3600 cm^–1^ range where rather narrow spectral features are observed. Here, deviations are affected by a slight IR frequency shift (<2 cm^–1^), perhaps due to incomplete warming up of the laser before the measurement. For instance, the blue trace (16 April) is visibly red-shifted. In conclusion, with only very minor deviations in both intensity and IR frequency, the repeatability and reproducibility of the current IRIS setup is excellent.

**Figure 3:**
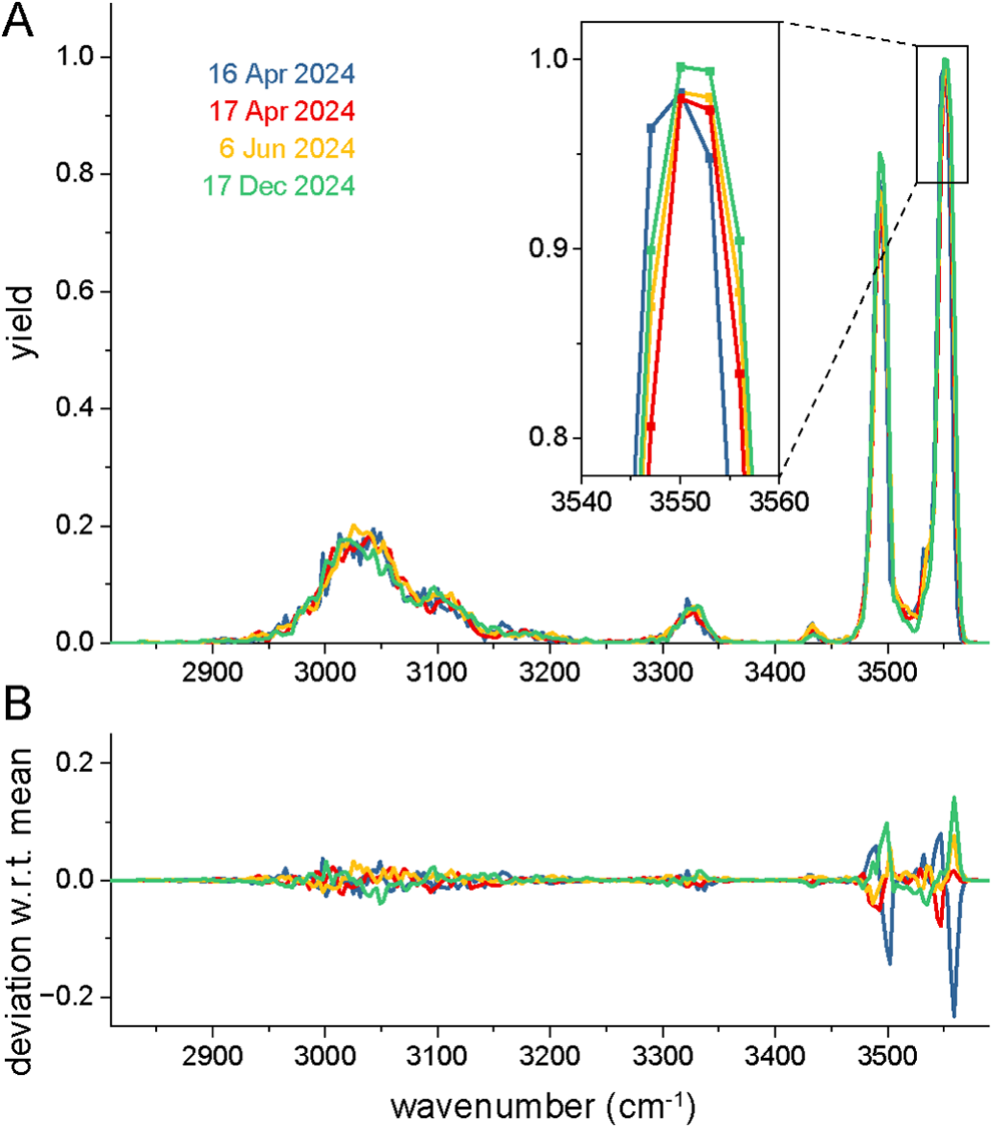
A: IRIS spectra of protonated tryptophan measured on different days. B: Deviation from the mean yield determined for the four spectra.

The second benchmark system is deprotonated *para*-coumaric acid ([PCA–H]^−^, *m*/*z* 163), an ion that is difficult to dissociate with currently available IR lasers. First IRMPD spectra were reported by Almasian et al.^48^ and used 75 pulses of a 10 Hz OPO (20 mJ/pulse in 6 ns) on a Fourier transform ion cyclotron resonance mass spectrometer (FT-ICR). To have sufficient on-resonance dissociation, the ions were irradiated for 20 ms with the output of a 30 W CO_2_ laser directly after each OPO pulse. Two OH stretching vibrations were observed at 3590 and 3650 cm^–1^ that could be assigned to phenoxide and carboxylate isomers, respectively. The relative abundance of the two isomers was shown to be strongly dependent on the electrospray solvent: protic solvents favor carboxylate and aprotic solvents favor phenoxide isomers. Experiments with the same OPO laser (but without additional CO_2_ laser) in a Bruker amaZon QIT were less successful: even upon reducing the helium pressure to its minimum (GC_He_ = 0%), IR dissociation yields were never above 0.1.^24,41^ Similar experiments with high repetition IR OPOs resulted in a substantial improvement:^41^ a 20 kHz (650 mW) OPO^41^ showed a dissociation yield of 0.15 at GC_He_ = 0% and <0.01 at GC_He_ = 20% for an irradiation time of 1 s.

We repeated these measurements with the current setup (30 kHz 2000 mW OPO and Bruker QIT) and the results are displayed in Figure 4. Clearly, the high power OPO provides substantially more dissociation than previously obtained with the 20 kHz (650 mW) OPO. For minimal helium, the band at 3590 cm^–1^ has a flat top, indicating that all ions of the absorbing isomer are dissociated. The observed yield of 0.75 then indicates that 75% of the ions are phenoxide isomer. The other band, due to the carboxylate isomer, shows a yield of 0.17, but without a flat top, indicating that not all ions are dissociated yet and longer irradiation times are needed to reach a maximum yield of 0.25. Nevertheless, this example of a difficult-to-dissociate ion clearly shows the advantage of IR lasers with high power.

**Figure 4:**
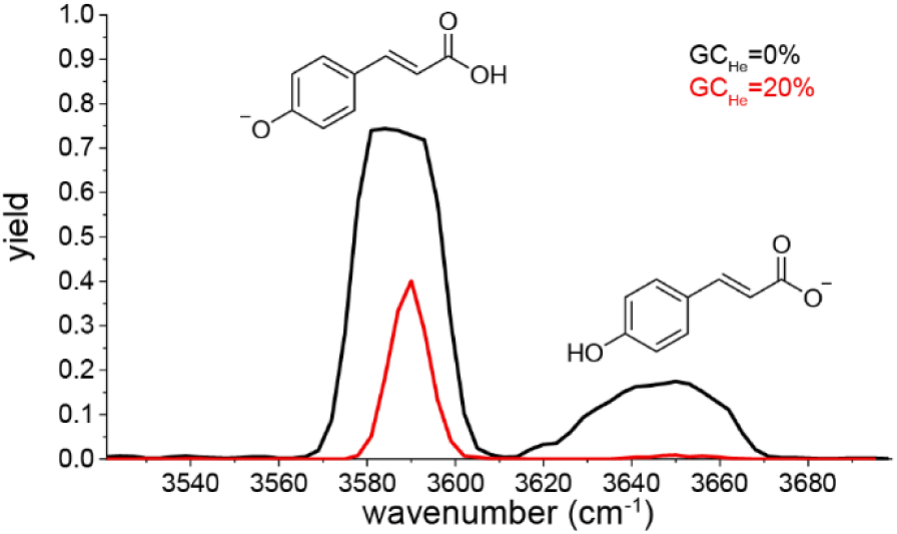
IR spectra of deprotonated para-coumaric acid (PCA, *m*/*z* 163) recorded at reduced helium pressure (GC_He_ = 20%, red trace) and without helium (GC_He_ = 0%, black trace) in the QIT. The ions were irradiated for 1 s with the 30 kHz 2 W OPO. The two bands observed at 3590 cm^–1^ and 3650 cm^–1^ correspond to the OH stretch vibration of the phenoxide and carboxylate isomer, respectively.

### Applications

A potential IRIS application area is the field of drug metabolite identification, an essential part of the drug discovery and development process, where one identifies the biotransformation products of drug molecules in the human body.^49,50^ Drug metabolism proceeds via a limited set of known reactions that either introduce a functional group in the drug, break a bond within the drug or link the drug molecule to an endogenous compound. Therefore, large parts of the metabolite structure are usually known (i.e., identical to the drug) and identification often involves determining the site of functionalization, cleavage or linkage.

One of the key advantages of using IRIS for molecular identification is that IR spectra, particularly when obtained in the gas phase, can be reliably calculated with quantum-chemical software packages. While molecular structure assignments based on predicted spectra may not always yield definitive conclusions, these calculations typically help to significantly narrow down the list of candidate structures. As a result, this approach substantially reduces the number of reference molecules that must be synthesized and minimizes the need for metabolite purification during various stages of the drug development lifecycle.

The determination of glucuronidation sites in drugs or their metabolites presents a significant challenge in metabolite identification by mass spectrometry.^51,52^ Glucuronidation is a process catalyzed by various uridine diphosphate (UDP) glucuronosyltransferases and consists of the transfer of a glucuronic acid to a functional group (hydroxyl, amine, thiol or carboxylic acid) of the substrate molecule. Due to its inability to form a structure-informative fragment, the most common phase II metabolic pathway is often difficult to determine if multiple glucuronidation sites are possible on a drug candidate.

We recorded the spectrum of a metabolite of the drug serotonin, protonated serotonin-O-glucuronide, *m*/*z* 353, shown in Figure 5A, and predicted its IR spectrum using density functional theory (DFT) at the B3LYP-D3/def2-TZVP level of theory for three possible glucuronidation sites of serotonin. DFT calculations provide the position and integrated intensity of the vibrational bands but not their band shape. To facilitate the comparison between experiment and theory, the stick spectra are broadened with a 10 cm^–1^ Gaussian line shape and displayed in Figure 5 panel B, C, and D.

**Figure 5:**
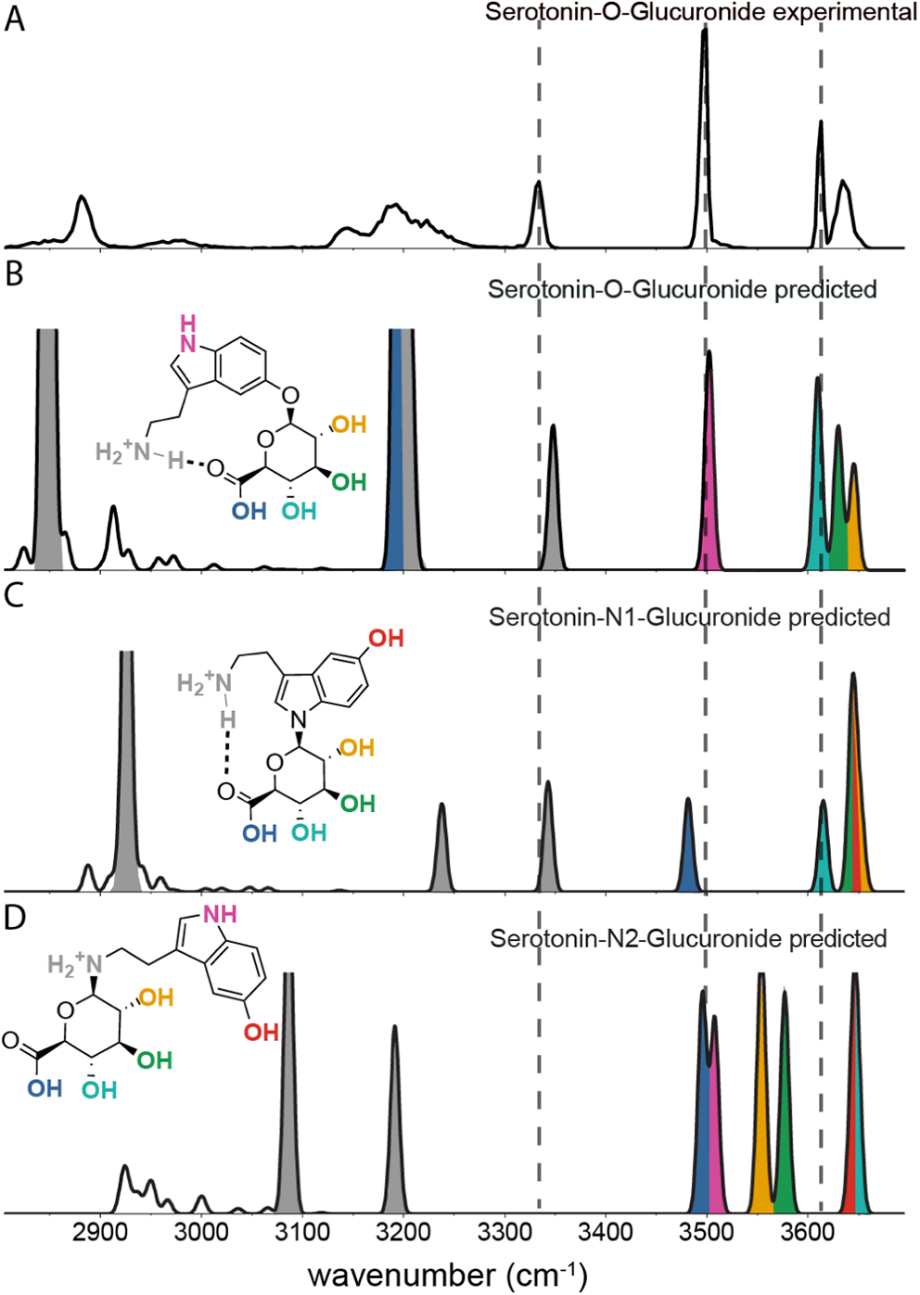
Molecular identification of serotonin glucuronide. A: Experimental spectrum of protonated serotonin-O-glucuronide. B, C, and D: Calculated spectra of, respectively, protonated serotonin-O-, -N1-, and -N2-glucuronide.

In the experimental spectrum, we observe the OH-stretching bands of the free hydroxyl-groups at 3600–3675 cm^–1^. The highest frequency peak at 3634 cm^–1^ is broader than the one at 3613 cm^–1^, suggesting that the feature contains multiple unresolved vibrational bands. Furthermore, we observe two sharp bands at 3499 and 3334 cm^–1^ from NH-stretching vibrations. To the lower wavenumber range of the spectrum, we observe broader IR bands corresponding to hydrogen bonded OH- and NH-stretches and CH-stretches.

The predictions are the most accurate for the free OH- and NH-stretches. We observe a clear match between the experimental spectrum and that calculated for the O-glucuronide: the bands of the free OH-groups of the glucuronide and the band at 3499 cm^–1^, originating from the aromatic NH-stretch, match both in position and relative intensity. The band at 3334 cm^–1^, originating from the asymmetric NH-stretch of the protonated primary amine is predicted too high in frequency, likely due to the hydrogen bond with the carboxylic oxygen that is not modeled well by the harmonic calculations. In the lower wavenumber region of the predicted spectrum, the OH-stretch vibration of the hydrogen-bonded carboxylic acid group (∼3194 cm^–1^) has an extremely high calculated intensity, which is indicative of a shared-proton motif and results in a spectrally broadened band with reduced peak intensity in the experimental spectrum.^53,54^ The same holds for the intense band predicted at 2848 cm^–1^, which is present in the experimental spectrum as a very broad low-intensity band, that is attributed to the vibration of the proton between the primary amine and the carboxylic oxygen.

The broad spectral bands due to hydrogen bonding guide the identification process, which we demonstrate here as we compare the experimental spectrum of the O-glucuronide with the calculated spectrum of the N1-glucuronide. In the 3300–3700 cm^–1^ range, the calculated band positions of N1-glucuronide are close to those experimentally found for O-glucuronide, although band intensities differ. In the 3100–3300 cm^–1^ region, however, the appearance of the broad bands in the experimental spectrum, while there are no calculated bands with unusual high intensity, reveals that the calculated N1-glucuronide spectrum does not match the experimental spectrum. Finally, the calculated N2-glucuronide spectrum is completely different over the full frequency range. Hence, the site of serotonin glucuronidation can be determined from its IR spectrum in the hydrogen stretching range.

A second example is the differentiation between different types of phase I oxidation products. During metabolism, amitriptyline is oxidized at the nitrogen, leading to amitriptyline N-oxide, or at an aliphatic site, yielding 10-hydroxy-amitriptyline.^55^

The IRIS spectra of both protonated hydroxylated reference species (*m*/*z* = 294) are displayed in Figure 6A,B. A striking observation is that the spectra are very distinct. The protonated 10-hydroxy form shows a narrow band at 3619 cm^–1^ corresponding to the free OH stretch vibration of the hydroxyl moiety. This band is absent in the spectrum of the N-oxide isomer, which instead features a broad band around 3300–3400 cm^–1^, indicating that the OH moiety is involved in strong hydrogen bonding leading to a shared proton motif giving rise to the observed band shape.

**Figure 6:**
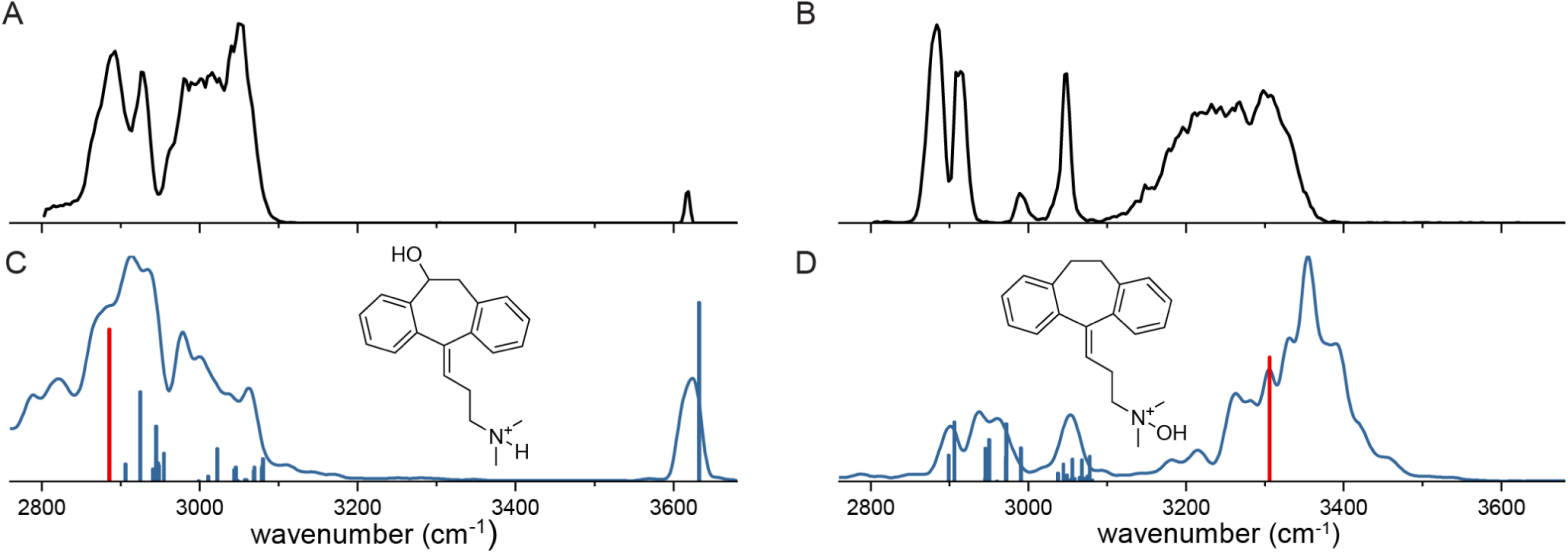
A and B: IRMPD spectrum of protonated 10-hydroxy amitriptyline and protonated N-oxide amitriptyline, respectively. C and D: BOMD calculated spectra (blue traces). Sticks: Scaled harmonic calculations. The red sticks are downscaled in intensity by a factor of 20.

Calculated DFT^56^ spectra for the two ions at the B3LYP-D4/def2-SVP level are displayed as the stick spectra in Figure 6C,D. The free OH stretch vibration in the 10-hydroxy-isomer is correctly predicted. For the N-oxide, a very intense band is calculated at 3306 cm^–1^ (red stick in Figure 6D, intensity scaled down by a factor of 20). The characteristically very high intensity is typical for vibrational modes that involve a shared proton motif. Indeed, visual inspection of the vibrational mode shows that the vibrational motion involves stretching of the proton from the NO toward the aromatic π-system. Note that the calculated spectrum of the protonated 10-hydroxy-system also exhibits a very intense band at 2885 cm^–1^, which is attributed to the NH-stretching mode, where the proton is strongly hydrogen bonded to the hydroxyl oxygen.

The dynamic behavior of the shared protons leading to broad band shapes cannot be captured with static DFT-based frequency calculations. More advanced Born–Oppenheimer Molecular dynamics^53,57,58^ (BOMD) calculations are necessary. Results of such BOMD modeled spectra are displayed as the blue curve in Figure 6C,D for the amitriptyline hydroxylated isomers and in Figure S1 for the serotonin-glucuronides. Although the BOMD calculations are clearly superior in predicting the band shapes, we note that this level of theory is not strictly necessary to distinguish these isomeric phase I product ions; standard DFT-based harmonic frequency calculations provide sufficient confidence for identification.

## Conclusion and Outlook

The availability of high-power infrared lasers and developments in the field of infrared ion spectroscopy brought the technique to the pharmaceutical industry. With a compact optomechanically stable setup we are able to measure IRIS spectra with high repeatability. The high power, high repetition laser allows for very high IR dissociation yields that have not been reported before. This allows one to detect low-intensity bands and record spectra for ions with high dissociation thresholds. In the accessible 3 μm spectral region, OH, NH and CH stretching vibrations dominate, making this tabletop IRIS setup attractive for the analysis of many classes of organic molecules. As demonstrated here, the setup has the potential to distinguish (positional) isomers of metabolites, which is often of critical importance in drug development. The use of density functional theory to predict IR spectra enables reference-free molecular identification. At first glance, a disadvantage of the 3 μm spectral range might be the appearance of broad spectral bands resulting from strongly hydrogen-bonded OH and NH groups, where the 0 K nature of (an)­harmonic frequency calculations fail to describe the dynamic behavior. In such calculations, bands evolving from the stretching motion of shared protons typically have unusually high intensities and are therefore easily recognized. Alternatively, Born–Oppenheimer molecular dynamics calculations can describe these broadened spectral features more precisely and can thereby validate the vibrational mode assignments. For the future, the development of high-power tabletop lasers capable of reaching the fingerprint region (5 to 20 μm) would greatly enhance the discrimination of positional isomers, such as formed upon oxidation of aromatic moieties within a drug molecule. With the introduction and demonstration of an IRIS setup in an industrial setting, we envision that IRIS will become a powerful addition to the analytical toolkit and find application in a wide range of fields, such as impurity analysis, forensics, and environmental sciences.

## Supplementary Material



## Notes

Methods for quantum chemical calculations and Born–Oppenheimer molecular dynamic calculations; additional calculated spectra for protonated serotonin glucuronides (PDF)

## References

[1] Wu Y., Pan L., Chen Z., Zheng Y., Diao X., Zhong D. (2021). Metabolite
Identification in the Preclinical and Clinical Phase of Drug Development. Curr. Drug Metab..

[2] Leclercq L., Cuyckens F., Mannens G. S. J., de Vries R., Timmerman P., Evans D. C. (2009). Which Human Metabolites
Have We MIST? Retrospective
AnalysisPractical Aspects, and Perspectives For Metabolite Identification
and Quantification in Pharmaceutical Development. Chem. Res. Toxicol..

[3] Zhu M., Zhang H., Humphreys W. G. (2011). Drug Metabolite Profiling and Identification
by High-resolution Mass Spectrometry. J. Biol.
Chem..

[4] Prakash C., Shaffer C. L., Nedderman A. (2007). Analytical strategies for identifying
drug metabolites. Mass Spectrometry Rev..

[5] Caceres-Cortes J., Reily M. D. (2010). NMR Spectroscopy
as a Tool to Close The Gap on Metabolite
Characterization Under MIST. Bioanalysis.

[6] Keun H. C., Beckonert O., Griffin J. L., Richter C., Moskau D., Lindon J. C., Nicholson J. K. (2002). Cryogenic Probe 13C NMR Spectroscopy
of Urine for Metabonomic Studies. Anal. Chem..

[7] Nikzad N., Rafiee M. (2024). Electrochemical study
of drug metabolism. Curr. Opin. Electrochem..

[8] Yao M., Tong N., Baghla R., Ruan Q. (2024). Advancing structural
elucidation of conjugation drug metabolites in metabolite profiling
with novel electron-activated dissociation. Rapid Commun. Mass Spectrom..

[9] Martens J., van Outersterp R. E., Vreeken R. J., Cuyckens F., Coene K. L. M., Engelke U. F., Kluijtmans L. A. J., Wevers R. A., Buydens L. M. C., Redlich B. (2020). Infrared ion spectroscopy: New opportunities
for small-molecule identification in mass spectrometry - A tutorial
perspective. Anal. Chim. Acta.

[10] van
Outersterp R. E., Engelke U. F. H., Merx J., Berden G., Paul M., Thomulka T., Berkessel A., Huigen M. C. D. G., Kluijtmans L. A. J., Mecinović J. (2021). Metabolite Identification Using Infrared Ion Spectroscopy—Novel
Biomarkers for Pyridoxine-Dependent Epilepsy. Anal. Chem..

[11] Abikhodr A. H., Ben Faleh A., Warnke S., Yatsyna V., Rizzo T. R. (2023). Identification
of human milk oligosaccharide positional isomers by combining IMS-CID-IMS
and cryogenic IR spectroscopy. Analyst.

[12] Martens J., Berden G., van Outersterp R. E., Kluijtmans L. A. J., Engelke U. F., van Karnebeek C. D. M., Wevers R. A., Oomens J. (2017). Molecular
identification in metabolomics using infrared ion spectroscopy. Sci. Rep..

[13] Ben
Faleh A., Warnke S., Van Wieringen T., Abikhodr A. H., Rizzo T. R. (2023). New Approach for the Identification
of Isobaric and Isomeric Metabolites. Anal.
Chem..

[14] Vink M. J. A., van Geenen F. A. M. G., Berden G., O’Riordan T. J. C., Howe P. W. A., Oomens J., Perry S. J., Martens J. (2022). Structural
Elucidation of Agrochemicals and Related Derivatives Using Infrared
Ion Spectroscopy. Environ. Sci. Technol..

[15] van
Outersterp R. E., Martens J., Berden G., Lubin A., Cuyckens F., Oomens J. (2023). Identification of Drug Metabolites
with Infrared Ion Spectroscopy – Application to Midazolam in
vitro Metabolism. Chem. Meth..

[16] Bell M. R., Tesler L. F., Polfer N. C. (2019). Cryogenic infrared
ion spectroscopy
for the structural elucidation of drug molecules: MDMA and its metabolites. Int. J. Mass Spectrom..

[17] Mucha E., González Flórez A. I., Marianski M., Thomas D. A., Hoffmann W., Struwe W. B., Hahm H. S., Gewinner S., Schöllkopf W., Seeberger P. H. (2017). Glycan Fingerprinting via Cold-Ion Infrared
Spectroscopy. Angew. Chem., Int. Ed..

[18] Schindler B., Laloy-Borgna G., Barnes L., Allouche A.-R., Bouju E., Dugas V., Demesmay C., Compagnon I. (2018). Online Separation
and Identification of Isomers Using Infrared Multiple Photon Dissociation
Ion Spectroscopy Coupled to Liquid Chromatography: Application to
the Analysis of Disaccharides Regio-Isomers and Monosaccharide Anomers. Anal. Chem..

[19] Grabarics M., Lettow M., Kirschbaum C., Greis K., Manz C., Pagel K. (2022). Mass Spectrometry-Based
Techniques to Elucidate the Sugar Code. Chem.
Rev..

[20] Yeni O., Ollivier S., Moge B., Ropartz D., Rogniaux H., Legentil L., Ferrières V., Compagnon I. (2023). Ring-Size
Memory of Galactose-Containing MS/MS Fragments: Application to the
Detection of Galactofuranose in Oligosaccharides and Their Sequencing. J. Am. Chem. Soc..

[21] Abikhodr A. H., Warnke S., Ben Faleh A., Rizzo T. R. (2024). Combining Liquid
Chromatography and Cryogenic IR Spectroscopy in Real Time for the
Analysis of Oligosaccharides. Anal. Chem..

[22] Neu V., Hoffmann W., Weiß T. D., Puhl M., Abikhodr A., Warnke S., Ben Faleh A., Klinck S., Pommer M., Kellner S. (2024). Validated Multimethod Approach for Full Characterization
of 2′-Fucosyl-d-lactose as an Industrially Produced Human Milk
Oligosaccharide. Anal. Chem..

[23] Houthuijs K. J., Berden G., Engelke U. F. H., Gautam V., Wishart D. S., Wevers R. A., Martens J., Oomens J. (2023). An *In Silico* Infrared Spectral Library
of Molecular Ions for Metabolite Identification. Anal. Chem..

[24] Martens J., Berden G., Gebhardt C. R., Oomens J. (2016). Infrared ion
spectroscopy
in a modified quadrupole ion trap mass spectrometer at the FELIX free
electron laser laboratory. Rev. Sci. Instrum..

[25] Vink M. J. A., Schermer J. J., Martens J., Buma W. J., Berden G., Oomens J. (2023). Characterization of
Solar Radiation-Induced Degradation
Products of the Plant Sunscreen Sinapoyl Malate. ACS Agric. Sci. Technol..

[26] Corinti D., Maccelli A., Crestoni M. E., Cesa S., Quaglio D., Botta B., Ingallina C., Mannina L., Tintaru A., Chiavarino B. (2019). IR ion spectroscopy in a combined approach
with MS/MS and IM-MS to discriminate epimeric anthocyanin glycosides
(cyanidin 3-O-glucoside and -galactoside). Int.
J. Mass Spectrom..

[27] Martens J., Berden G., Bentlage H., Coene K. L. M., Engelke U. F., Wishart D., van Scherpenzeel M., Kluijtmans L. A. J., Wevers R. A., Oomens J. (2018). Unraveling the unknown areas of the
human metabolome: the role of infrared ion spectroscopy. J. Inherited Metab. Dis..

[28] van
Geenen F. A. M. G., Kranenburg R. F., van Asten A. C., Martens J., Oomens J., Berden G. (2021). Isomer-Specific Two-Color
Double-Resonance IR2MS3 Ion Spectroscopy Using a Single Laser: Application
in the Identification of Novel Psychoactive Substances. Anal. Chem..

[29] Vink M. J. A., Alarcan J., Martens J., Buma W. J., Braeuning A., Berden G., Oomens J. (2024). Structural
Elucidation of Agrochemical
Metabolic Transformation Products Based on Infrared Ion Spectroscopy
to Improve In Silico Toxicity Assessment. Chem.
Res. Toxicol..

[30] Houthuijs K. J., Horn M., Vughs D., Martens J., Brunner A. M., Oomens J., Berden G. (2023). Identification
of organic micro-pollutants
in surface water using MS-based infrared ion spectroscopy. Chemosphere.

[31] Kranenburg R. F., van Geenen F. A. M. G., Berden G., Oomens J., Martens J., van Asten A. C. (2020). Mass-Spectrometry-Based Identification of Synthetic
Drug Isomers Using Infrared Ion Spectroscopy. Anal. Chem..

[32] Seo J., Hoffmann W., Warnke S., Huang X., Gewinner S., Schöllkopf W., Bowers M. T., von Helden G., Pagel K. (2017). An infrared spectroscopy approach to follow β-sheet formation
in peptide amyloid assemblies. Nat. Chem..

[33] Maitre P., Scuderi D., Corinti D., Chiavarino B., Crestoni M. E., Fornarini S. (2020). Applications
of Infrared Multiple
Photon Dissociation (IRMPD) to the Detection of Posttranslational
Modifications. Chem. Rev..

[34] Sinha R. K., Maître P., Piccirillo S., Chiavarino B., Crestoni M. E., Fornarini S. (2010). Cysteine radical
cation: A distonic
structure probed by gas phase IR spectroscopy. Phys. Chem. Chem. Phys..

[35] Roithová J., Gray A., Andris E., Jašík J., Gerlich D. (2016). Helium Tagging Infrared Photodissociation
Spectroscopy
of Reactive Ions. Acc. Chem. Res..

[36] Nosenko Y., Menges F., Riehn C., Niedner-Schatteburg G. (2013). Investigation
by two-color IR dissociation spectroscopy of Hoogsteen-type binding
in a metalated nucleobase pair mimic. Phys.
Chem. Chem. Phys..

[37] Penna T. C., Cervi G., Rodrigues-Oliveira A.
F., Yamada B. D., Lima R. Z. C., Menegon J. J., Bastos E. L., Correra T. C. (2020). Development
of a photoinduced fragmentation ion trap for infrared multiple photon
dissociation spectroscopy. Rapid Commun. Mass
Spectrom..

[38] Bakels S., Daly S., Doğan B., Baerenfaenger M., Commandeur J., Rijs A. M. (2024). Probing High-Order Transient Oligomers
Using Ion Mobility Mass Spectrometry Coupled with Infrared Action
Spectroscopy. Anal. Chem..

[39] Harrilal C. P., Garimella S. V. B., Norheim R. V., Ibrahim Y. M. (2025). Development of a
Platform for High-Resolution Ion Mobility Separations Coupled with
Messenger Tagging Infrared Spectroscopy for High-Precision Structural
Characterizations. Anal. Chem..

[40] Polfer N. C. (2011). Infrared
multiple photon dissociation spectroscopy of trapped ions. Chem. Soc. Rev..

[41] van
Outersterp R. E., Martens J., Peremans A., Lamard L., Cuyckens F., Oomens J., Berden G. (2021). Evaluation of table-top
lasers for routine infrared ion spectroscopy in the analytical laboratory. Analyst.

[42] Berden G., Derksen M., Houthuijs K. J., Martens J., Oomens J. (2019). An automatic
variable laser attenuator for IRMPD spectroscopy and analysis of power-dependence
in fragmentation spectra. Int. J. Mass Spectrom..

[43] Mino W. K., Gulyuz K., Wang D., Stedwell C. N., Polfer N. C. (2011). Gas-Phase
Structure and Dissociation Chemistry of Protonated Tryptophan Elucidated
by Infrared Multiple-Photon Dissociation Spectroscopy. J. Phys. Chem. Lett..

[44] Hamlow L. A., Zhu Y., Devereaux Z. J., Cunningham N. A., Berden G., Oomens J., Rodgers M. T. (2018). Modified
Quadrupole Ion Trap Mass Spectrometer for
Infrared Ion Spectroscopy: Application to Protonated Thiated Uridines. J. Am. Soc. Mass Spectrom..

[45] Yeni O., Schindler B., Moge B., Compagnon I. (2022). Rapid IRMPD
(InfraRed multiple photon dissociation) analysis for glycomics. Analyst.

[46] Lucas B., Grégoire G., Lemaire J., Maître P., Glotin F., Schermann J. P., Desfrançois C. (2005). Infrared multiphoton
dissociation spectroscopy of protonated N-acetyl-alanine and alanyl-histidine. Int. J. Mass Spectrom..

[47] Prell J. S., O’Brien J. T., Williams E. R. (2010). IRPD Spectroscopy and Ensemble Measurements:
Effects of Different Data Acquisition and Analysis Methods. J. Am. Soc. Mass Spectrom..

[48] Almasian M., Grzetic J., van Maurik J., Steill J. D., Berden G., Ingemann S., Buma W. J., Oomens J. (2012). Non-Equilibrium Isomer
Distribution of the Gas-Phase Photoactive Yellow Protein Chromophore. J. Phys. Chem. Lett..

[49] Chen Y., Monshouwer M., Fitch W. L. (2007). Analytical Tools and Approaches for
Metabolite Identification in Early Drug Discovery. Pharm. Res..

[50] Lu W., Xu Y., Zhao Y., Cen X. (2015). Emerging Technologies,
Recent Developments,
and Novel Applications for Drug Metabolite Identification. Curr. Drug Metab..

[51] Yuan L., Xu X. S., Ji Q. C. (2020). Challenges
and Recommendations in
Developing LC–MS/MS Bioanalytical Assays of Labile Glucuronides
and Parent Compounds in the Presence of Glucuronide Metabolites. Bioanalysis.

[52] Guo Y., Shah A., Oh E., Chowdhury S. K., Zhu X. (2022). Determination of Acyl-, O-, and N-Glucuronide Using Chemical Derivatization
Coupled with Liquid Chromatography–High-Resolution Mass Spectrometry. Drug Metab. Dispos..

[53] Martínez-Haya B., Avilés-Moreno J. R., Gámez F., Martens J., Oomens J., Berden G. (2023). Correlated
proton dynamics
in hydrogen bonding networks: the benchmark case of 3-hydroxyglutaric
acid. Phys. Chem. Chem. Phys..

[54] Corinti D., Berden G., Oomens J., Martinez-Haya B., Fornarini S., Crestoni M. E. (2024). IRMPD spectroscopy
of deprotonated
selenocysteine - The 21st proteinogenic amino acid. Int. J. Mass Spectrom..

[55] Rudorfer M. V., Potter W. Z. (1999). Metabolism of Tricyclic Antidepressants. Cell. Mol. Neurobiol..

[56] Neese F. (2022). Software update:
The ORCA program system—Version 5.0. Wiley Interdiscip. Rev.: Comput. Mol. Sci..

[57] Brehm M., Thomas M., Gehrke S., Kirchner B. (2020). TRAVIS—A free
analyzer for trajectories from molecular simulation. J. Chem. Phys..

[58] Franzke Y. J., Holzer C., Andersen J. H., Begušić T., Bruder F., Coriani S., Della Sala F., Fabiano E., Fedotov D. A., Fürst S. (2023). TURBOMOLE: Today and Tomorrow. J. Chem. Theory
Comput..

