# Supplementary material for: Development of a Robust Platform for Infrared Ion Spectroscopy: A New Addition to the Analytical Toolkit for Enhanced Metabolite Structure Elucidation
Source: Anal Chem. 2025 Jul 31;97(31):17216–23. doi: 10.1021/acs.analchem.5c03593 (PMC12355478; doi:10.1021/acs.analchem.5c03593)
Supplement: Supplementary file 1 [file ac5c03593_si_001.pdf]

Supporting information

Development of a Robust Platform for Infrared Ion Spectroscopy: A New Addition to the Analytical Toolkit for Enhanced Metabolite Structure Elucidation

Teun van Wieringen[§,1], Arnaud Lubin[§,2], Rianne van Outersterp[1], Jonathan Martens[1], Eric van Beelen[3], Jos Oomens[1], Filip Cuyckens[2], Giel Berden[*,1].

[1] Radboud University, Institute for Molecules and Materials, FELIX Laboratory, Toernooiveld 7, 6525 ED Nijmegen, The Netherlands

[2] Drug Metabolism & Pharmacokinetics, Johnson & Johnson, Turnhoutseweg 30, B-2340 Beerse, Belgium

[3] Bruker Daltonics GmbH & Co. KG, Fahrenheitstraße 4, D-28359 Bremen, Germany

[§] T.W. and A.L. contributed equally to this paper.
[*] Corresponding author: Giel Berden – FELIX Laboratory, Institute for Molecules and Materials, Radboud University, Toernooiveld 7, NL-6525 ED Nijmegen, The Netherlands; Phone: +31 243653951; Email: g.berden@science.ru.nl

Table of Contents

Quantum Chemistry – Serotonin Glucuronides S2

Born-Oppenheimer Molecular Dynamics S2

Figure S1: Comparison of the experimental IRMPD spectrum of protonated Serotonin-O-Glucuronide with the BOMD calculated spectra for Serotonin-O-Glucuronide and Serotonin-N1-Gluruconide. S2

References S3

Quantum Chemistry – Serotonin Glucuronides

Calculations of the serotonin glucuronides have been performed with ORCA version 6.0.1[1]. Protonated conformers have been generated as previously described and optimized using the HF-3c method.[2] After selection of the conformers up to a free energy of 40 kJ/mol, they are further optimized using r2scan-3c. The resulting conformers were selected again up to 25 kJ/mol and optimized at B3LYP-D3 / def2-TZVP after which the vibrational spectrum is calculated using the harmonic approximation. A single point energy calculation is done at MP2 / 6-31++G(d,p), combined with the thermal contribution to yield the free energy, used to rank the conformers. The lowest energy conformer is evaluated for each of the isomer. Frequencies are scaled by a linear scaling factor of 0.96.

Born-Oppenheimer Molecular Dynamics

Molecular dynamics calculations are done using Turbomole 7.8 [3]. The input geometry was optimized at the same level of theory as the dynamics. Four duplicate molecular dynamics runs were done using different random initial velocities as chosen by the mdprep module of Turbomole corresponding to a kinetic energy temperature of 350 K. 40 000 molecular dynamics steps were done with time steps of 20 $\hbar/E_h$ (~0.48 fs) at 350 K using a Nosé-Hoover thermostat with a relaxation time of 400 $\hbar/E_h$. The initial velocities were chosen such that there is no linear or angular momentum around the center of mass and the linear and angular momentum is set to zero every 56 steps to prevent drift of the center of mass by rerunning mdprep. For each time step, the dipole as computed by Turbomole is saved to a file. The IR spectrum was calculated from the Fourier transform of the autocorrelation of the dipole moments using the Travis[4] software package, in which the dipole moments are loaded. The first 5000 steps are skipped in the analysis. The calculated IR spectrum for the four independent runs are combined to give the final predicted spectrum. BOMD frequencies are scaled using a linear scaling factor of 0.96.

The amitriptyline isomers are calculated at B3LYP-D4 / def2-SVP level of theory and 40 000 md steps were taken. The Serotonin-Glucuronides are calculated at r2scan-D4 / def2-SVP level of theory and 60 000 md steps were taken.

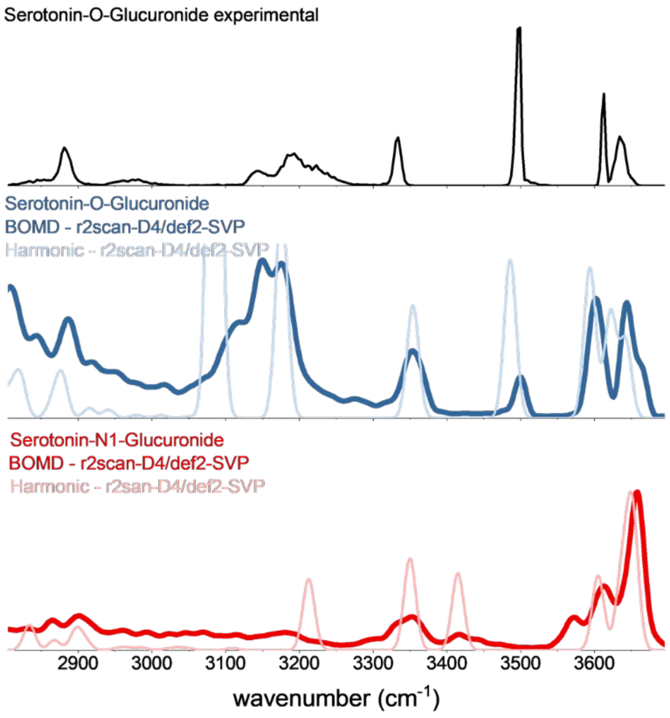

Fig S1. Comparison of the experimental IRMPD spectrum of protonated Serotonin-O-Glucuronide (top) with the BOMD calculated spectra for Serotonin-O-Glucuronide and Serotonin-N1-Gluruconide (in middle and bottom panel respectively). The harmonic calculated spectra, calculated on the same level of theory are added to give insight in the calculation.

References

(1) Neese, F. Software update: The ORCA program system—Version 5.0. *WIREs Computational Molecular Science* **2022**, *12* (5), e1606. DOI: 10.1002/wcms.1606.
(2) Houthuijs, K. J.; Berden, G.; Engelke, U. F. H.; Gautam, V.; Wishart, D. S.; Wevers, R. A.; Martens, J.; Oomens, J. An *In Silico* Infrared Spectral Library of Molecular Ions for Metabolite Identification. *Analytical Chemistry* **2023**, *95* (23), 8998-9005. DOI: 10.1021/acs.analchem.3c01078.
(3) Franzke, Y. J.; Holzer, C.; Andersen, J. H.; Begušić, T.; Bruder, F.; Coriani, S.; Della Sala, F.; Fabiano, E.; Fedotov, D. A.; Fürst, S.; et al. TURBOMOLE: Today and Tomorrow. *Journal of Chemical Theory and Computation* **2023**, *19* (20), 6859-6890. DOI: 10.1021/acs.jctc.3c00347.
(4) Brehm, M.; Thomas, M.; Gehrke, S.; Kirchner, B. TRAVIS—A free analyzer for trajectories from molecular simulation. *The Journal of Chemical Physics* **2020**, *152* (16), 164105. DOI: 10.1063/5.0005078.
